# Identification of the BRAF V600E mutation in gastroenteropancreatic neuroendocrine tumors

**DOI:** 10.18632/oncotarget.6602

**Published:** 2015-12-14

**Authors:** Charny Park, Sang Yun Ha, Seung Tae Kim, Hee Cheol Kim, Jin Seok Heo, Young Suk Park, Gregory Lauwers, Jeeyun Lee, Kyoung-Mee Kim

**Affiliations:** ^1^ Department of Pathology and Translational Genomics, Samsung Medical Center, Sungkyunkwan University School of Medicine, Seoul, Korea; ^2^ Division of Hematology-Oncology, Department of Medicine, Samsung Medical Center, Sungkyunkwan University School of Medicine, Seoul, Korea; ^3^ Department of Surgery, Samsung Medical Center, Sungkyunkwan University School of Medicine, Seoul, Korea; ^4^ Department of Pathology, Massachusetts General Hospital, Boston, MA, USA

**Keywords:** neuroendocrine tumors, BRAFV600E mutation, pazopanib

## Abstract

Genomic profiles of gastroenteropancreatic neuroendocrine tumors (GEP-NETs) are still insufficiently understood, and the genetic alterations associated with drug responses have not been studied. Here, we performed whole exome sequencing of 12 GEP-NETs from patients enrolled in a nonrandomized, open-labeled, single-center phase II study for pazopanib, and integrated our results with previously published results on pancreas (*n* = 12) and small intestine NETs (*n* = 50). The mean numbers of somatic mutations in each case varied widely from 20 to 4682. Among 12 GEP-NETs, eight showed mutations of more than one cancer-related gene, including *TP53, CNBD1, RB1, APC, BCOR, BRAF, CTNNB1, EGFR, EP300, ERBB3, KDM6A, KRAS, MGA, MLL3, PTEN, RASA1, SMARCB1, SPEN, TBC1D12*, and *VHL*. TP53 was recurrently mutated in three cases, whereas *CNBD1* and *RB1* mutations were identified in two cases. Three GEP-NET patients with *TP53* mutations demonstrated a durable response and one small intestinal grade (G) 1 NET patient with *BRAF* V600E mutation showed progression after pazopanib treatment. We found *BRAF* V600E (G1 NET from rectum and two G3 NETs from colon) and *BRAF* G593S (G2 NET from pancreas) missense mutations (9.1%) in an independent cohort of 44 GEP-NETs from the rectum (*n* = 26), colon (*n* = 7), pancreas (*n* = 4), small intestine (*n* = 3), stomach (*n* = 3) and appendix (*n* = 1) by Sanger sequencing. All tumor specimens were obtained before chemotherapy. In conclusion, *BRAF* V600E mutation is likely to result in resistance to pazopanib but may be a potentianally actionable mutation in metastatic GEP-NETs patients.

## INTRODUCTION

Gastroenteropancreatic neuroendocrine tumors (GEP-NETs) are relatively rare tumors accounting for about 0.5% of all human cancers [1–6]. Their incidence is significantly increasing based on data from recent population-based studies, and this phenomenon is explained by increased awareness of the disease entity and increased detection by advanced diagnostic modalities [1–6]. However, there has been no significant improvement in clinical outcome over the same period based on UK and US databases [7].

Recently, the FDA approved a few targeted agents for pancreatic NETs including sunitinib, a multi-tyrosine kinase inhibitor, and everolimus, an inhibitor of the PI3K-Akt-mTOR signal pathway [8]. In pancreatic NET, sunitinib was compared to a placebo in a phase III trial of 171 pancreatic NET patients, and the median progression-free survival was significantly prolonged in the sunitinib arm (11.4 versus 5.5 months) [9]. Based on these data, sunitinib was approved in the US for the treatment of progressive, well-differentiated pancreatic NET. In randomized controlled trials, everolimus demonstrated a 65% decrease in the risk for tumor progression in pancreatic NETs [7] and a 23% decrease in patients with non-pancreatic NETs [9]. In an analysis of 159 patients with NETs, everolimus showed a response rate of 7.7% with a progression-free survival of 12 months [10].

We recently conducted a phase II trial for pazopanib in metastatic GEP-NET patients [11]. Our phase II study demonstrated an objective response rate of 18.9% (7 of 37, 95% CI 8.0 – 35.2) and a disease control rate (CR + confirmed PR + stable disease) of 75.7% (28 of 37, 95% CI, 58.8 – 88.2) in metastatic GEP-NETs. Through this trial, we observed that a small subset of NET patients responded to pazopanib for > 6 months. Recently, pancreatic NETs were characterized as having recurrent somatic mutations in *MEN1*, *DAXX*, *ATRX, TSC*, and *PTEN* on the basis of exome sequencing of 10 pancreatic NETs [12]. Small intestinal NETs showed recurrent somatic mutations and deletions in *CDKN1B* by whole exome and whole genome sequencing of 50 small intestinal NETs [13]. However, in another study of 48 small intestinal NETs by exome sequencing, recurrent mutations were not identified. Rather, 197 single nucleotide variations in a preponderance of cancer-related genes were identified; 33% of small intestinal NET patients showed PIK3/Akt/mTOR pathway alteration, and 72% had therapeutically actionable genomic alterations [13]. The understanding of genomic profiles in GEP-NETs is still incomplete and the genetic alterations associated with drug responses have not been extensively studied. In this study, we performed whole exome sequencing of 12 GEP-NETs from patients enrolled in a nonrandomized, open-labeled, single-center phase II study of pazopanib [11].

## RESULTS

### Genomic profiling identifies the BRAF V600E mutation in pazopanib non-responder and the TP53 mutation in pazopanib responder patients

**Figure 1:**
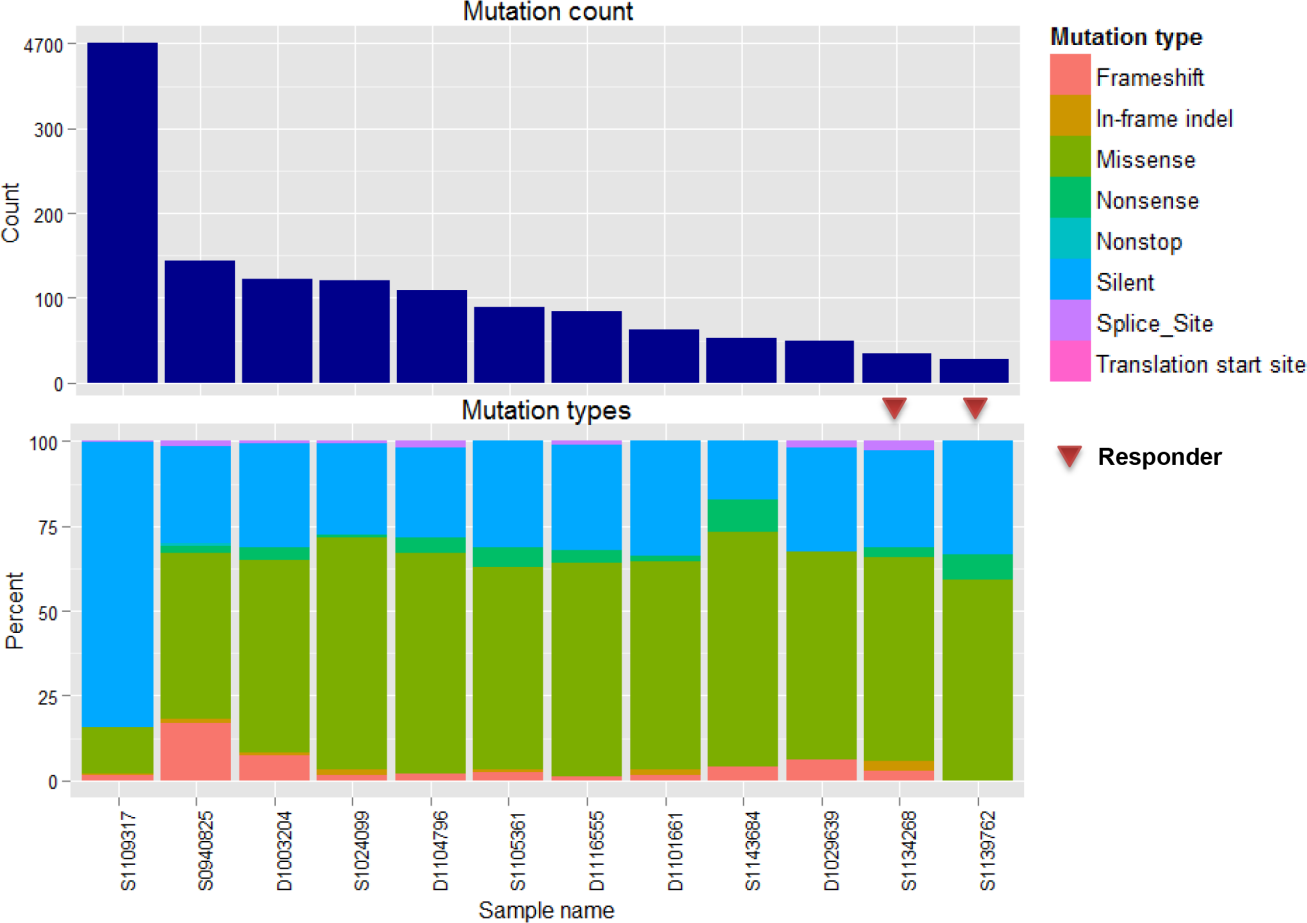
Mutations in 12 NET samples (**A** and **B)** show total counts and a percentage bar plot according to mutation type.

**Figure 2:**
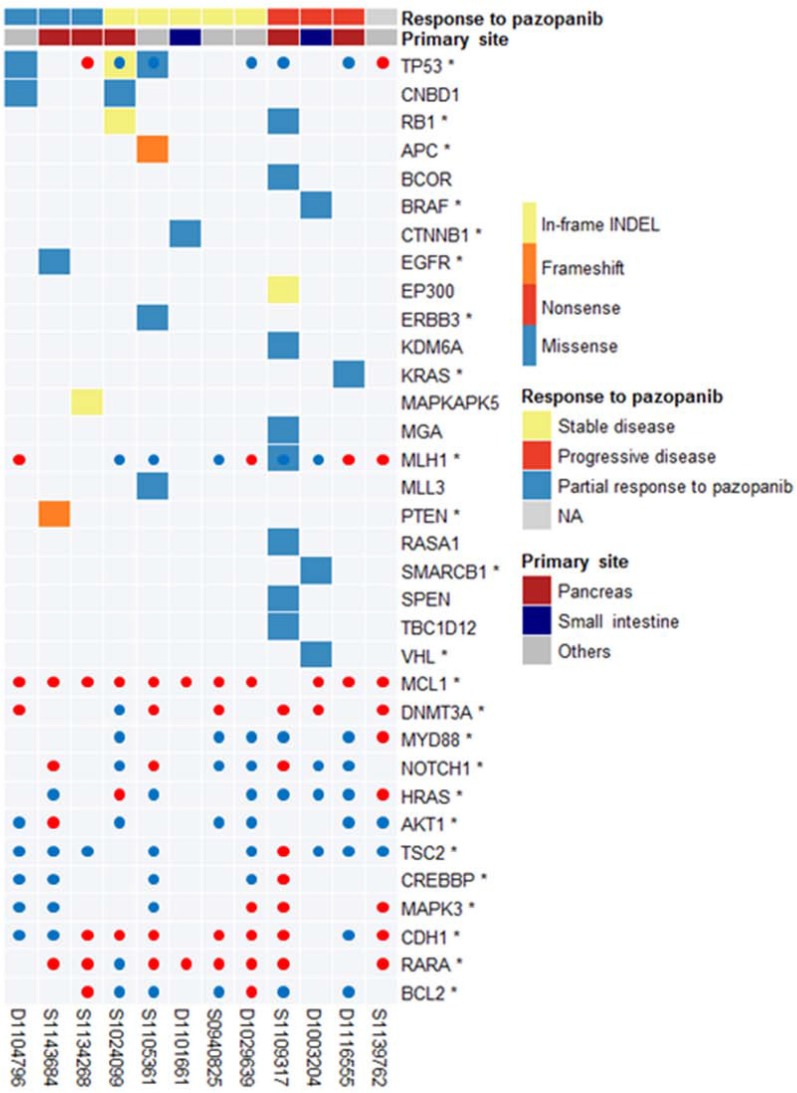
Landscape of cancer-related mutations found in 12 GEP-NETs

The mean number of somatic mutations varied widely from 20 to 4682, and the mutation counts for each case are shown in Figure 1. One case with 4682 somatic mutations showed a mutation in *MLH1* and additional missense mutations (*ATR*, *PARP2*, *RBBP8*, and *RIF1*) and splice site (*XPC*) mutations in DNA repair-related genes, and was thus classified as having a “hypermutated” phenotype. Among 12 samples, eight showed mutations of more than one of the cancer-related genes (*TP53, CNBD1, RB1, APC, BCOR, BRAF, CTNNB1, EGFR, EP300, ERBB3, KDM6A, KRAS, MGA, MLL3, PTEN, RASA1, SMARCB1, SPEN, TBC1D12*, and *VHL*) described by Lawrence et al. [24] (Figure 2). *TP53* was recurrently mutated in three cases, whereas *CNBD1* and *RB1* mutations were identified in two cases. In our data set, we found the presence of *BRAF* V600E mutation in one primary NET from the small intestine, which was further confirmed by Sanger direct sequencing. To exclude the possibility of the occurrence of mixed adenocarcinoma, neuroendocrine features, or both, we performed an independent pathology review in terms of architecture, tumor grade, and chromogranin, synaptophysin, and CD56 immunoreactivity by IHC (Supplementary Table 1). All of the pathological features corresponded to GEP-NET, rather than mixed adenocarcinoma with neuroendocrine features.

**Figure 3:**
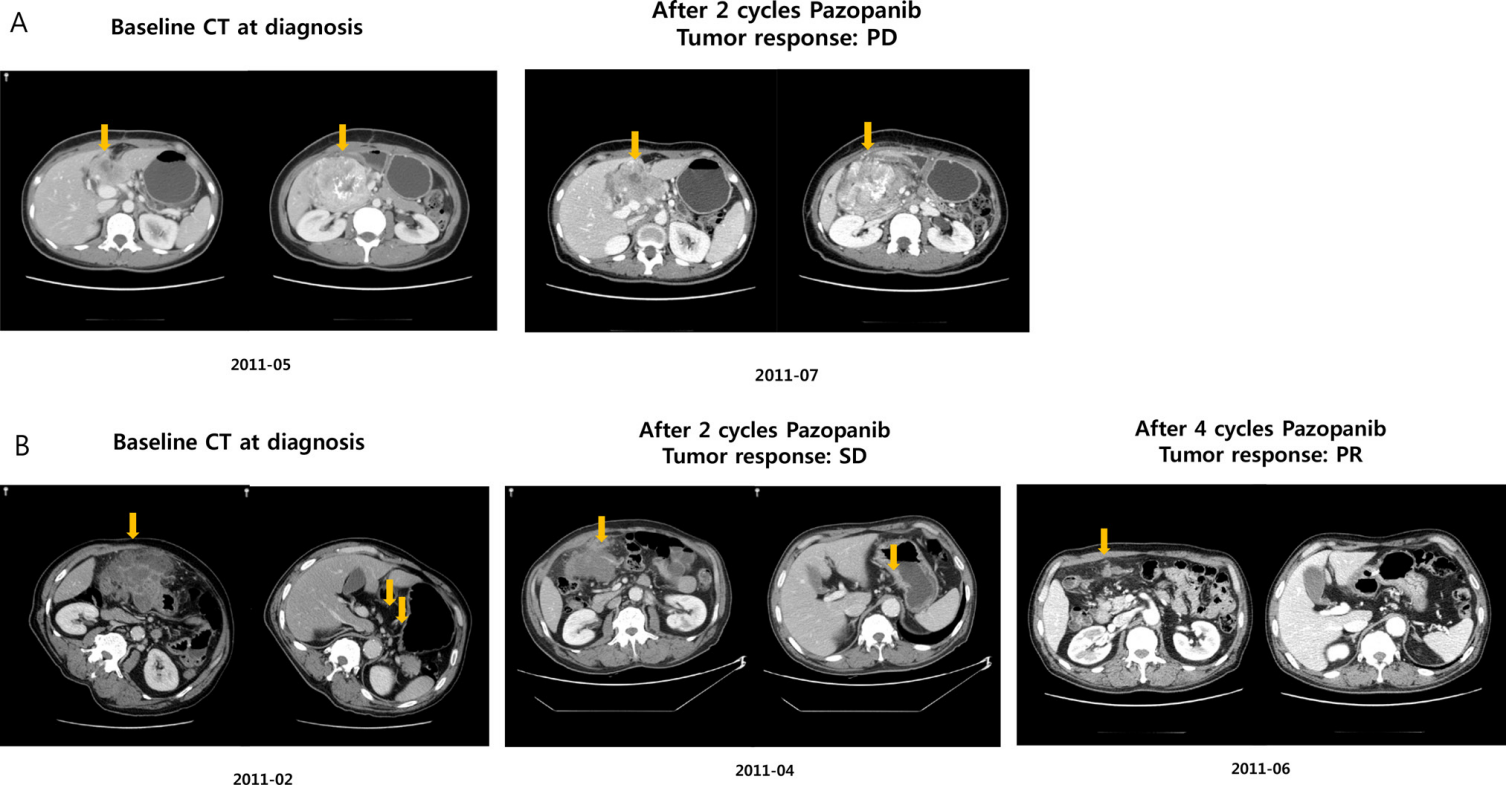
Response to pazopanib (**A**) BRAF V600E mutant; (**B**) TP53 mutant NET patient.

The patient with *BRAF V600E* mutation was 52 years old and had a metastatic grade 1 neuroendocrine tumor. The primary mass originated from the duodenum and had metastasized to multiple and distant lymph nodes at diagnosis. The patient received capecitabine and oxaliplatin as a palliative first-line treatment. After disease progression following the first-line therapy, the patient was enrolled in the pazopanib clinical trial. Before starting pazopanib, mutational profiles of the primary tumor tissue were evaluated. After two cycles of pazopanib therapy, the follow-up computed tomography (CT) scan revealed tumor growth corresponding to disease progression based on RECIST 1.1 criteria (Figure 3A). Hence, this small intestinal NET patient with *BRAF* V600E mutation showed tumor progression after pazopanib treatment, although at the time of clinical trial enrollment, the genomic information was not available to the clinician because this trial was not a genome-selected trial.

We also identified the *TP53* mutation in a patient with a dramatic response to pazopanib. The patient had a grade 3 neuroendocrine carcinoma with gastric primary tumor location and extensive abdominal lymph node and peritoneal seeding nodule involvement. After failing to respond to cytotoxic chemotherapy, the patient was treated with pazopanib. After 2 cycles of pazopanib, the patient presented stable disease per the RECIST 1.1 criteria (Figure 3B). At 4 months, CT evaluation (after 4 cycles) revealed definite radiologic tumor shrinkage corresponding to a partial response based on the RECIST 1.1 criteria (Figure 3B), which lasted for > 6 months.

### BRAF mutations in an independent cohort

**Figure 4:**
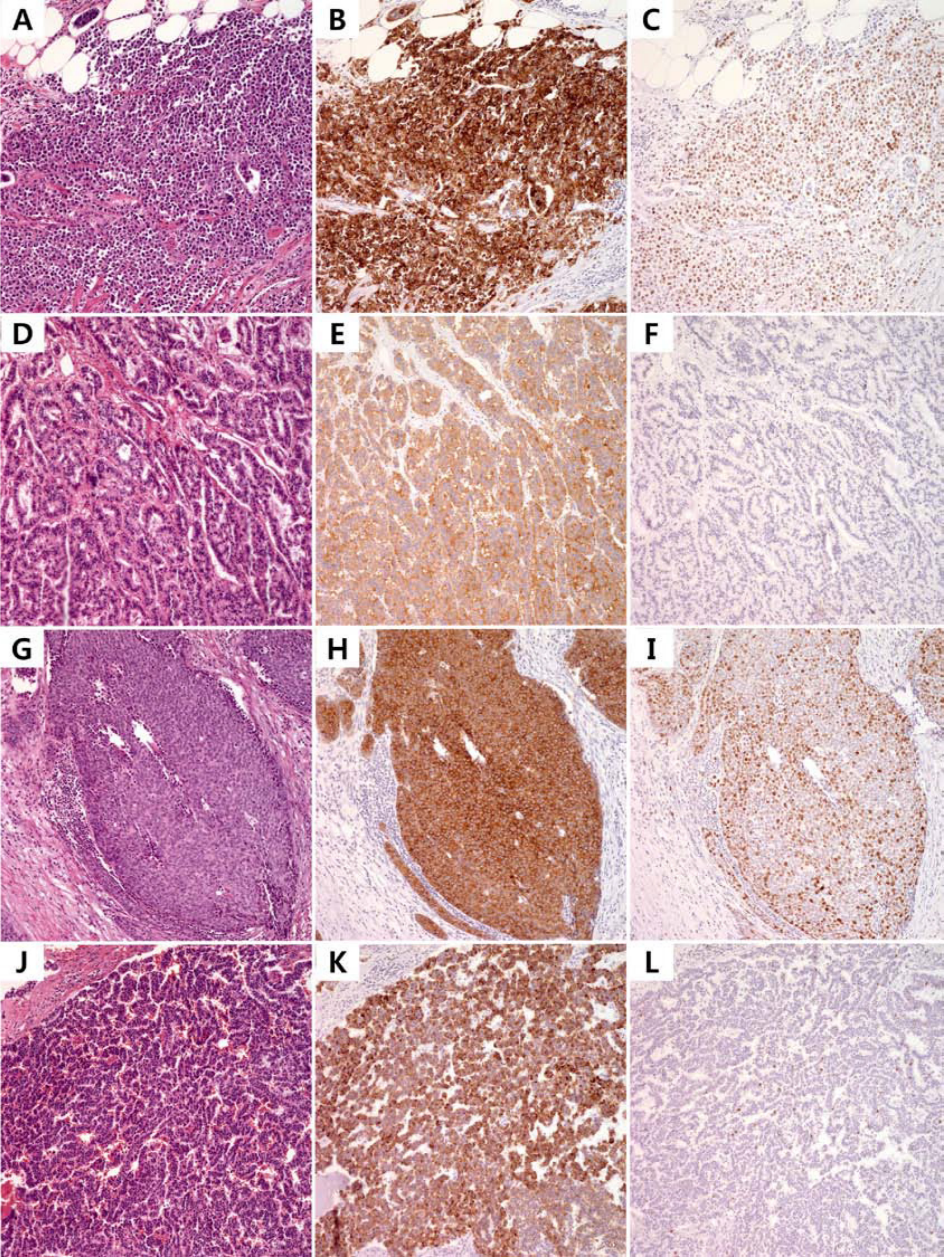
Photomicrograph of BRAF-mutant NETs from independent cohort Pathologic findings of V600E mutant small intestinal G3 NET (**A**), V600E mutant rectal G1 NET (**D**), V600E mutant sigmoid colon G3 NET (**G**) and G593S mutant G2 pancreas NET (**J**) and corresponding immunohistochemistry for synaptophysin (**B, E, H**) and chromogranin (**K**) with Ki-67 (**C, F, I, L**).

Next, we surveyed the presence of the *BRAF* V600E mutation in an independent cohort of 44 GEP-NET patients. We included GEP-NETs from the rectum (*n* = 26 consisting 19 G1, 4 G2 and 3 G3), colon (*n* = 7 consisting 6 G3 and one G1), pancreas (*n* = 4, G2), small intestine (*n* = 3 consisting one G3 and 2 G2), stomach (*n* = 3 consisting 2 G3 and one G1) and appendix (*n* = 1, adenocarcinoid). We found *BRAF* V600E (G1 NET from rectum and two G3 NETs from colon) and *BRAF* G593S (G2 NET from pancreas) missense mutations (9.1%) in an independent cohort of 44 GEP-NETs by Sanger sequencing. The cohort consisted of GEP-NET from rectum (*n* = 27), colon (*n* = 6), pancreas (*n* = 4), small intestine (*n* = 3), stomach (*n* = 3) and appendix (*n* = 1). All G1 and G2 NETs were positive for synaptophysin while rectal NETs and G3 NETs were negative for chromogranin (Figure 4). The pathology and immunohistochemical staining of the four NETs with BRAF V600E or G593S mutation are shown in Figure 4 (G1, 1-cm rectal NET; G2, 8.5 cm pancreatic NET; G3, 6.5 cm colon NET; and G3, 3.2 cm colon NET).

### Copy number variations in GEP-NET

The copy number profile of Korean GEP-NETs clearly demonstrates a difference in CNV pattern for each primary organ (Supplementary Figure 1). The copy number of pancreatic NET relatively fluctuated interchromosomally. In contrast, there was no concordance of arm-level copy number in small intestinal NET (Supplementary Table 2). Our copy number results are similar to those of a previous small intestinal NET study [13]. Moreover, arm-level amplification of 19q13.31 (*q* value, 0.49) frequently occurred in the pancreas, i.e., in three of four amplification samples (Supplementary Tables 1 and 2). Fourteen CNV genes (*MCL1, DNMT3A, MLJ1, MYD88, NOTCH1, HRAS, AKT1, TSC2, CREBBP, MARK3, CDH1, TP53, RARA*, and *BCL2*) that may potentially be actionable are illustrated in Figure 2.

### MAPKAPK5 mutation in an exceptional responder to pazopanib

Whole exome sequencing results in two cases showed an exceptional response to pazopanib. In these two cases, we could not find mutations in any of the 272 recurrently mutated genes. Furthermore, we investigated the mutation status of genes in four pathways (hsa04010, MAPK signaling pathway; hsa04020, calcium signaling pathway; hsa04060, cytokine-cytokine receptor interaction; hsa05200, pathways in cancer) involved in the primary mechanism of action of pazopanib. We found a novel p.I16fs *MAPKAPK5* mutation involved in the MAPK signaling pathway in a patient with pancreatic NET (Supplementary Figure 2).

### Integrative analysis of Korean GEP-NETs with pancreatic NETs and small intestinal NETs from previously published data

As our GEP-NETs included various primary organs, we integrated our results with previously published results for pancreatic and small intestinal NETs [12, 13]. Most of the mutated genes did not overlap among the three datasets, suggesting different genomic alterations according to the primary location of the tumor (Supplementary Figure 3). In this analysis, we found 272 recurrently mutated genes irrespective of the organ, and we used those recurrently mutated genes to construct a gene interaction network.

**Figure 5:**
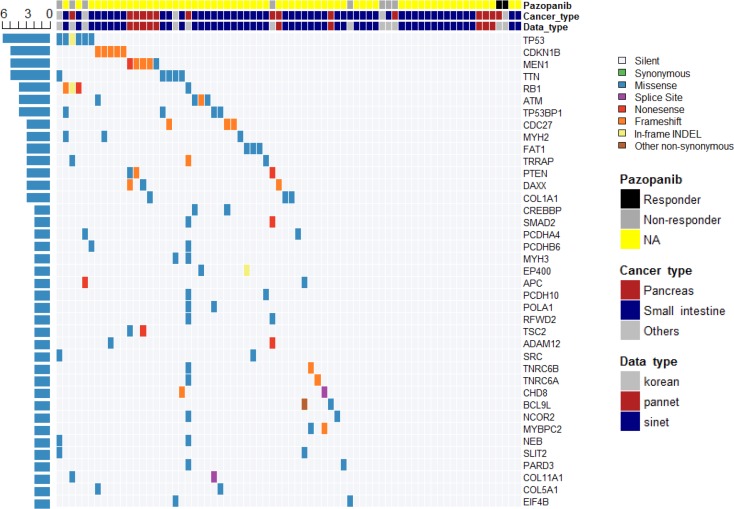
Overall mutational profile of all GEP-NETs revealed by whole exome and whole genome studies

Finally, a network of 65 genes and 89 interactions was generated after genes that did not pass the GSEA test were trimmed (Supplementary Figure 4 and Table 2). Genes related to cell cycle, Wnt signaling, E2F transcription factor network, DNA damage, p53 pathway, EGFR signaling, FGFR signaling, ERBB2 signaling, PDGFR signaling, and PI3K-Akt signaling pathways were mostly associated with these genes (Table 2). *TP53* was the gene with the most recurrent mutations, as mutations were observed in 6 cases in various organs (Figure 5). *CDKN1B* mutation was found in five cases of small intestinal NET, whereas *MEN1* mutation was found in five cases of pancreatic NET. Mutations of *RB1*, *ATM*, and *TP53BP1* were each identified in four cases. Overall, 46 of 72 samples (64%) had at least one mutation in at least one cancer-related gene [24]. The overall mutational profile of all GEP-NETs, including ours, is presented in Figure 5.

**Table 1:** Patient characteristics

ID	Sex	ECOG	Histology		Sites of metastasis	Best response	Age
D1003204	2	1	G1	1	liver, LN, ovary	PD	48
D1029639	1	1	G1	1	liver, lung, adrenal	SD	46
D1101661	1	1	G3	2	liver, LN	SD	46
D1104796	1	1	G1	2	liver, LN, peritoneal seeding	PR	65
D1116555	1	1	G1	1	liver, LN	PD	61
S0929052	1	1	G2	1	liver	SD	70
S0940825	2	1	G2	1	liver, LN	SD	53
S1024099	1	1	G3	2	liver, LN	SD	59
S1105361	1	1	G3	2	liver	SD	72
S1109317	2	1	G3	2	liver	PD	71
S1134268	1	1	G3	2	liver	PR	19
S1139762	1	0	G2	1	liver, lung	SD	64
S1143684	2	1	G2	1	liver	PR	69

**Table 2:** Pathway analysis

Gene set	Ratio of protein in gene det	Number of proteins in gene set	Proteins from network	FDR	Nodes
Cell cycle(K)	0.0127	124	7	2.00E-04	TP53, RB1, CREBBP, SMAD2, CDC27, ATM, CDKN1B
Wnt signaling pathway(P)	0.0279	272	10	2.50E-04	PCDHA4, PCDHB6, MYH3, MYH2, TP53, EP400, FAT1, APC, CREBBP, PCDH10
E2F transcription factor network(N)	0.007	68	6	3.33E-04	RB1, POLA1, TRRAP, CREBBP, ATM, CDKN1B
Direct p53 effectors(N)	0.0135	132	8	5.00E-04	PTEN, TP53, RB1, RFWD2, TRRAP, APC, CREBBP, TSC2
DNA Damage/Telomere Stress Induced Senescence(R)	0.0027	26	5	1.00E-03	TP53, RB1, EP400, ATM, CDKN1B
regulation of transcriptional activity by pml(B)	0.0011	11	3	1.00E-03	DAXX, TP53, RB1
p53 signaling pathway(K)	0.007	68	5	1.71E-03	PTEN, TP53, RFWD2, ATM, TSC2
Signaling by EGFR(R)	0.0176	172	7	2.13E-03	PTEN, ADAM12, SRC, TNRC6B, TNRC6A, CDKN1B, TSC2
Pre-NOTCH Expression and Processing(R)	0.0039	38	4	2.89E-03	TP53, TNRC6B, TNRC6A, CREBBP
p53 pathway(P)	0.0045	44	4	4.55E-03	PTEN, TP53, CREBBP, ATM
Signaling by SCF-KIT(R)	0.014	137	6	4.67E-03	PTEN, SRC, TNRC6B, TNRC6A, CDKN1B, TSC2
TCF dependent signaling in response to WNT(R)	0.0153	149	6	4.67E-03	MEN1, TRRAP, CHD8, BCL9L, APC, CREBBP
HTLV-I infection(K)	0.0267	260	8	4.70E-03	TP53, RB1, TRRAP, APC, CREBBP, SMAD2, CDC27, ATM
Prostate cancer(K)	0.0091	89	5	4.71E-03	PTEN, TP53, RB1, CREBBP, CDKN1B
Notch-mediated HES/HEY network(N)	0.0047	46	4	4.77E-03	RB1, CREBBP, CDKN1B, NCOR2
Hepatitis B(K)	0.015	146	6	4.81E-03	PTEN, TP53, RB1, SRC, CREBBP, CDKN1B
PIP3 activates AKT signaling(R)	0.0096	94	5	4.94E-03	PTEN, TNRC6B, TNRC6A, CDKN1B, TSC2
Muscle contraction(R)	0.005	49	4	5.13E-03	MYBPC2, TTN, MYH3, NEB
Signaling by FGFR(R)	0.0159	155	6	5.35E-03	PTEN, SRC, TNRC6B, TNRC6A, CDKN1B, TSC2
Signaling by ERBB2(R)	0.0159	155	6	5.35E-03	PTEN, SRC, TNRC6B, TNRC6A, CDKN1B, TSC2
p53 pathway(N)	0.0058	57	4	6.00E-03	DAXX, TP53, CREBBP, ATM
p53 pathway feedback loops 2(P)	0.0025	24	3	6.36E-03	PTEN, TP53, ATM
Glypican 1 network(N)	0.0026	25	3	7.04E-03	SLIT2, SRC, SMAD2
Signaling by PDGF(R)	0.0179	175	6	8.17E-03	PTEN, SRC, TNRC6B, TNRC6A, CDKN1B, TSC2
Cell Cycle Checkpoints(R)	0.0119	116	5	8.40E-03	TP53, RFWD2, CDC27, ATM, CDKN1B
Signaling by TGF-beta Receptor Complex(R)	0.0072	70	4	9.93E-03	MEN1, PARD3, SMAD2, NCOR2
Oncogene Induced Senescence(R)	0.0031	30	3	1.01E-02	TP53, TNRC6B, TNRC6A
PI3K-Akt signaling pathway(K)	0.0356	347	8	1.04E-02	PTEN, COL11A1, TP53, COL1A1, COL5A1, EIF4B, CDKN1B, TSC2
Adherens junction(K)	0.0075	73	4	1.09E-02	PARD3, SRC, CREBBP, SMAD2
ATM pathway(N)	0.0035	34	3	1.23E-02	RFWD2, TP53BP1, ATM
FoxO signaling pathway(K)	0.0136	133	5	1.25E-02	PTEN, CREBBP, SMAD2, ATM, CDKN1B

## DISCUSSION

In this study, we performed whole-exome sequencing of 12 GEP-NETs from patients enrolled in a nonrandomized, phase II study of pazopanib and constructed a gene interaction network based on recurrently mutated genes with sequencing data from 72 GEP-NETs from three datasets [12, 13]. *TP53* (*n* = 6), *CDKN1B* (*n* = 5), *MEN1* (*n* = 5), *RB1* (*n* = 4), *ATM* (*n* = 4), and *TP53BP1* (*n* = 4) were frequently mutated genes. *CDK1B* mutation was identified only in small intestinal NET, whereas *MEN1* mutation was identified in pancreatic NET. These genes are related to the cell cycle, Wnt, E2F transcription factor, and DNA damage pathways. Overall, 46 of 72 samples (64%) had at least one mutation in cancer-related genes.

Importantly, we found one *BRAF* V600E mutation in small intestinal NET that did not respond to pazopanib and further confirmed that 3 (6.8%) of 44 GEP-NET patients harbored the *BRAF* V600E mutation. *BRAF* V600E is an actionable mutation in melanoma with significantly prolonged survival when a BRAF inhibitor is administered in patients with this mutation [25], but it is not actionable in colorectal adenocarcinoma. To exclude the possibility of mixed histology of adenocarcinoma and NET, we performed an independent pathology review and confirmed that all of the NET cases were not adenocarcinoma with neuroendocrine differentiation. Hence, *BRAF* V600E is a novel mutation found in NET that could potentially confer clinical benefit in this subset of patients. *BRAF* V600E mutations have been reported as oncogenic mutation or resistant mutation to drugs in melanoma [26, 27] and thyroid cancer [28], GIST [29], hairy cell leukemia [30], multiple myeloma [31], and pediatric metanephric tumors [32]. The clinical implications of the *BRAF* V600E mutation in metastatic GEP-NET should thus be evaluated in clinical trials.

Three GEP-NET patients with *TP53* mutations demonstrated a durable response to pazopanib, either as PR or achievement of stable disease (Figure 2). The tumor suppressor gene *TP53* is mutated in many cancer types, and various TP53 mutations (missense, frameshift [fs], or nonsense [*], leading to gain or loss of function) have been identified during tumorigenesis and metastasis [26]. Cancer cells with mutated *TP53* have accelerated tumor growth associated with increased VEGF expression and neovascularization [27], which represents an important survival pathway [7, 8], resulting in a therapeutic advantage of anti-angiogenesis inhibitors in *TP53* mutant cancer patients [28]. Although our findings should be confirmed in larger patient cohort, we postulate that GEP-NET patients with *TP53* mutation may have enhanced angiogenesis that may be treated using an anti-angiogenesis inhibitor, such as pazopanib.

Only a few reports have described the sequencing data of GEP-NETs, and most of them were performed for NETs from a specific organ. Jiao et al. [12] performed exome sequencing in 10 pancreatic NETs and found recurrently mutated genes as follows: *MEN1* (*n* = 5), *DAXX* (*n* = 3), *PTEN* (*n* = 2), and *TSC2* (*n* = 2). In a validation set of 68 cases, *MEN1* was mutated in 44% cases, and *DAXX* and *ATRX*, which interacts with *DAXX* to form a chromatin remodeling complex, were mutated in 25% and 18% cases, respectively, with a mutually exclusive pattern. Another important finding was that at least one gene involved in the mTOR pathway, such as *TSC2* or *PTEN*, was mutated in 14% of cases. This finding is consistent with observed clinical responses in pancreatic NET patients with the recently approved mTOR inhibitor everolimus [8]. Banck et al. [13] performed exome sequencing of 48 small intestinal NETs and identified 197 protein-altering somatic single nucleotide variations with a preponderance of cancer-related genes such as *FGFR2*, *MEN1*, *HOOK3*, *EZH2*, *MLF1*, *CARD11*, *VHL*, *NONO*, and *SMAD1*. However, most of these mutations were not recurrently identified. Using an integrated approach combining mutational and copy number data, Banck et al. found that 33% of small intestinal NET patients showed PIK3/Akt/mTOR pathway alteration and 72% had therapeutically actionable genomic alterations. Francis et al. [13] identified mutations of 1,230 genes by whole-exome and whole-genome sequencing of 50 small intestinal NETs. Approximately 90% of mutations were not recurrently mutated, and only *CDKN1B* was recurrently mutated in 10% of patients. Moreover, only a small number of mutated genes overlapped with previously reported small intestinal NETs or pancreatic NETs, suggesting different genomic alterations according to the primary location of the tumor or that many of the observed mutations were passenger and not driver mutations.

In our data sets where our results were combined with previously published results [12, 13], we confirmed that most of the mutated genes did not overlap among the three datasets, including ours. Therefore, we constructed a gene interaction network of 65 genes and 89 interactions using 272 recurrently mutated genes. As a result, traditional cancer-associated pathways, such as cell cycle, Wnt signaling pathway, E2F transcription factor network, DNA damage, p53 pathway, EGFR signaling, FGFR signaling, ERBB2 signaling, PDGFR signaling, and PI3K-Akt signaling pathway were mostly associated with the 65 genes. Notably, 46 of 72 samples (64%) showed at least one mutation of these 65 genes, suggesting that about two-thirds of GEP-NET patients may have a benefit from a drug targeting these pathways.

In this study, we found that a novel *MAPKAPK5* mutation involved in the MAPK signaling pathway affected the mechanism of action of pazopanib in a pazopanib responder. *MAPKAPK5* is known to be involved in tumor suppression, angiogenesis, and cytoskeletal remodeling through interaction with a variety of substrates and is associated with neurological processes, including neurosecretion [33]. However, *MAPKAPK5* mutations have not been documented in GEP-NET. Our results will facilitate the identification of biomarkers for the pazopanib response and will form the basis for a further large-scale genomic study.

The limitation of this study is that it was performed in a relatively small number of samples from various primary organs. To overcome this limitation, we tried to integrate our results with previously published data of pancreatic and small intestinal NETs. To our knowledge, the current study is the first using NET samples from patients enrolled in a clinical trial to identify genetic alterations associated with drug responses.

## MATERIALS AND METHODS

### Patient characteristics and sample preparation

We performed whole exome sequencing of 12 GEP-NET tumor specimens from patients enrolled in a nonrandomized, open-labeled, single-center phase II study after obtaining written informed consent, and the samples were processed using protocols approved by the Institutional Review Boards [11]. We extracted DNA from fresh tumor tissue (*n* = 2) and formalin-fixed paraffin-embedded tumor tissue (*n* = 10) as previously described [14]. The primary sites of GEP-NETs included rectum (*n* = 3), pancreas (*n* = 4), small intestine (*n* = 2), stomach (*n* = 1), and unknown primary sites (*n* = 2), and all the cases had hepatic metastasis at presentation. An independent pathologic review by an expert gastrointestinal pathologist (G.L.) is summarized in Supplementary Table 1. The histological grade was categorized as follows: carcinoid tumors and well differentiated NETs were classified as Grade 1 tumors, atypical carcinoid and well-differentiated neuroendocrine carcinomas were classified as Grade 2 tumors, and poorly differentiated neuroendocrine carcinomas were classified as Grade 3 tumors [15]. For validation of the BRAF mutation in an independent cohort, we extracted DNA from 44 primary GEP-NETs (> 1cm in size or > Grade 2) and Sanger-sequenced them as previously described [16]. All research involving human participants have been approved by SMC Institutional Review Board (IRB), and all clinical investigation has been conducted according to the principles expressed in the Declaration of Helsinki.

### Whole exome sequencing (WES) and data analysis

Sequencing data were generated using a protocol that has been detailed previously [13, 17]. Briefly, exonic regions were captured using the Agilent V2 capture probe set and sequenced by 76-bp paired-end reads using an Illumina HiSeq2000 instrument. A median of 129,621,217 total reads was generated for each sample, 97.72–99.28% reads of which were aligned to the target exome using the Burrows-Wheeler Aligner (BWA) [15], resulting in a median coverage of each base of 100X.

Downstream sequencing analysis was performed as previously described [13, 17]. Before mutation and indel calling, sequencing reads were locally realigned to improve the detection of indels and decrease the number of false-positive SNVs caused by misaligned reads, particularly at the 3′ end as previously described [13, 17]. For mutation detection, > 14 reads in the tumors and > 8 reads in the normal samples were necessary to call candidate somatic base substitutions, and indels were detected using MuTect [18]. Germline mutations were detected using the UnifiedGenotyper [19]. All somatic mutations were manually reviewed and visually confirmed using the Integrated Genomics Viewer (http://www.broadinstitute.org/igv/). Copy number variation (CNV) was analyzed by GISTIC 2.0 [20], and we investigated GEP-NET CNV genes from drug targets defined in a previous study [21].

### Mutational gene interaction network and gene set enrichment analysis

To investigate the genetic characteristics of GEP-NET, we integrated our mutation profiles with previously published data for pancreatic NETs (*n* = 10) [12] and small intestinal NETs (*n* = 50) [13]. To compose the gene set, silent mutations were eliminated and then recurrently mutated genes (mutations in greater than or equal to 2) were selected. As a result, 136 genes were entered as a gene set.

The interaction network was constructed from the input gene set by ReactomeFI of Cytoscape [22, 23] [PMID: 20482850, PMID: 14597658]. Gene set enrichment analysis (GSEA) for the reactome pathway was performed within a cutoff FDR of ≤ 0.01. Finally, the interaction network was trimmed to only include genes that passed the GSEA and first neighbors of GSEA genes.

## SUPPLEMENTARY MATERIALS FIGURES AND TABLE


