# Supplementary material for: Identification of the BRAF V600E mutation in gastroenteropancreatic neuroendocrine tumors
Source: Oncotarget. 2015 Dec 14;7(4):4024–35. doi: 10.18632/oncotarget.6602 (PMC4826187; doi:10.18632/oncotarget.6602)
Supplement: Supplementary file 1 [file oncotarget-07-4024-s001.pdf]

Identification of the BRAF V600E mutation in gastroenteropancreatic neuroendocrine tumors

Supplementary Materials

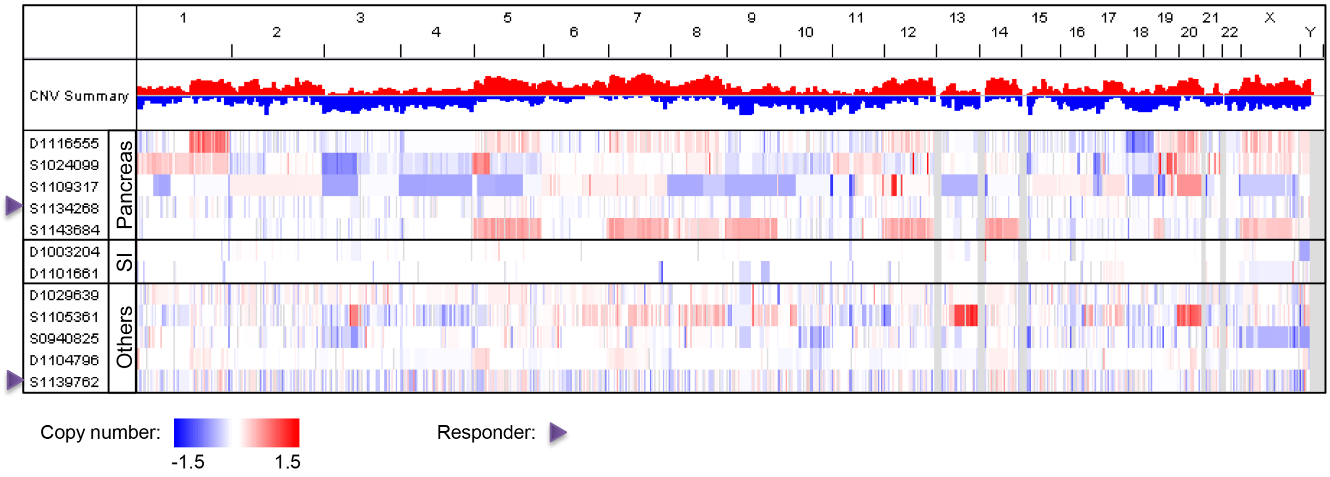

Supplementary Figure S1: Copy number analysis.

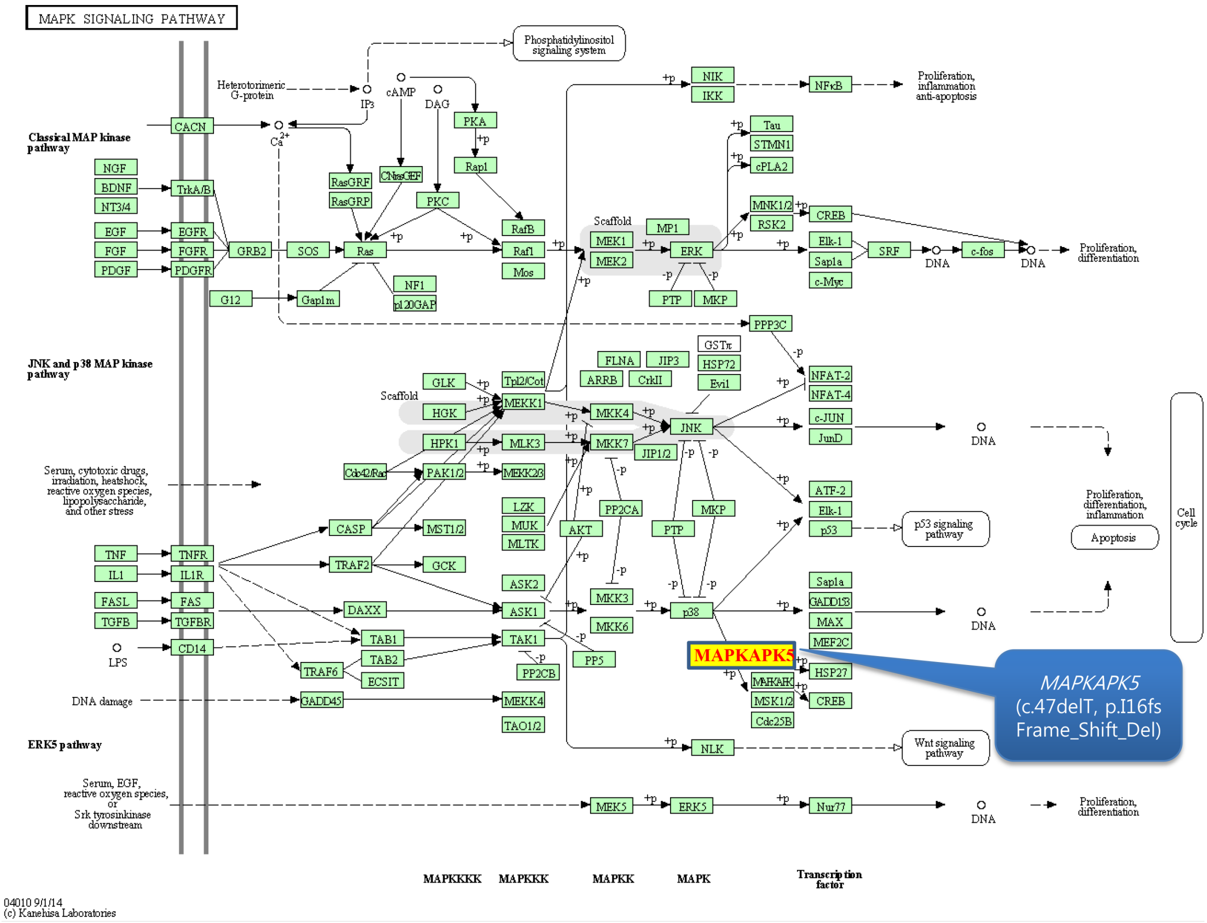

Supplementary Figure S2: MAPK signaling pathway. A novel *MAPKAPK5* mutation involved in MAPK signaling pathway was identified in a patient with pancreatic NET.

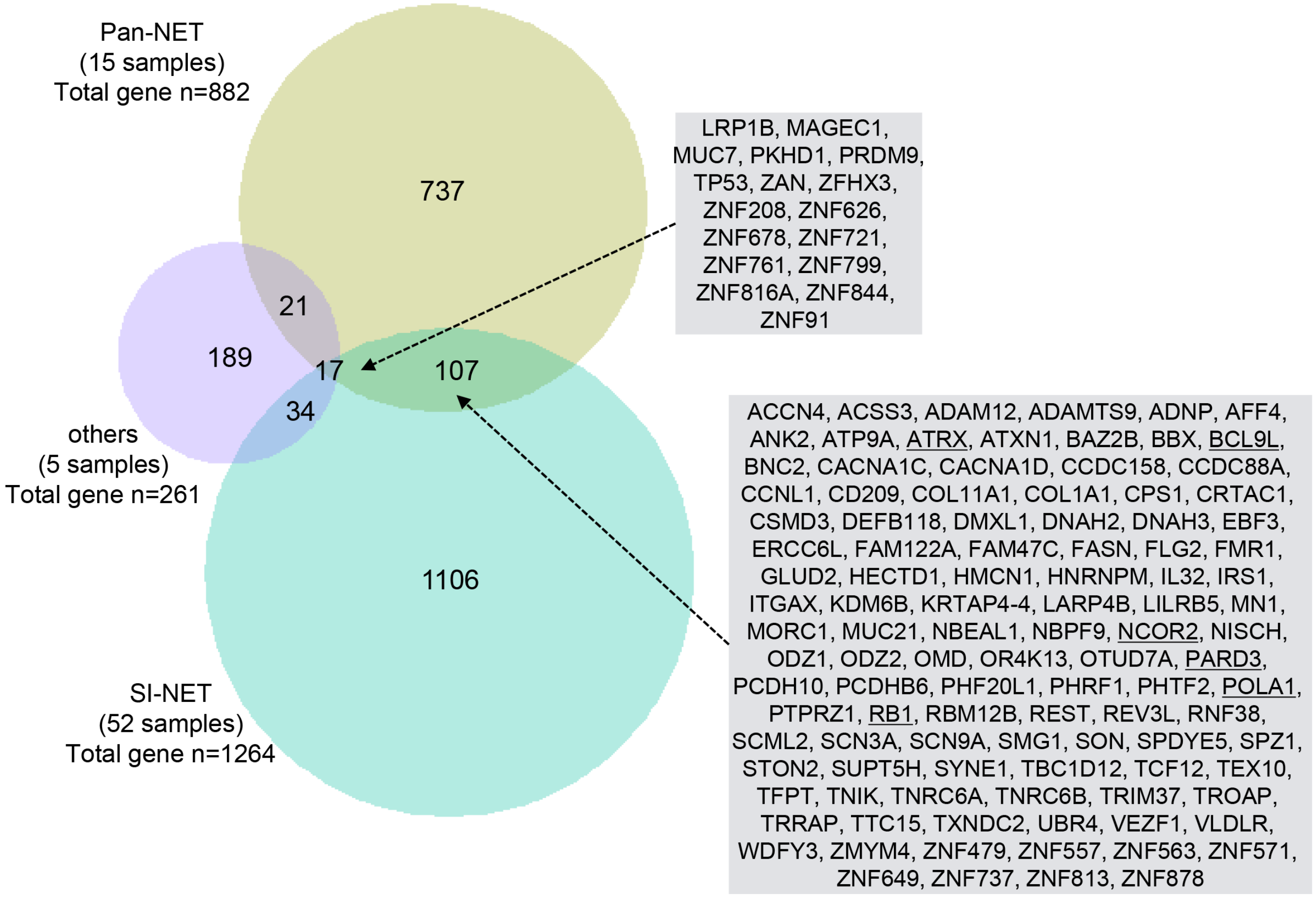

Supplementary Figure S3: Venn diagram of mutated genes identified from three NET studies, including ours.

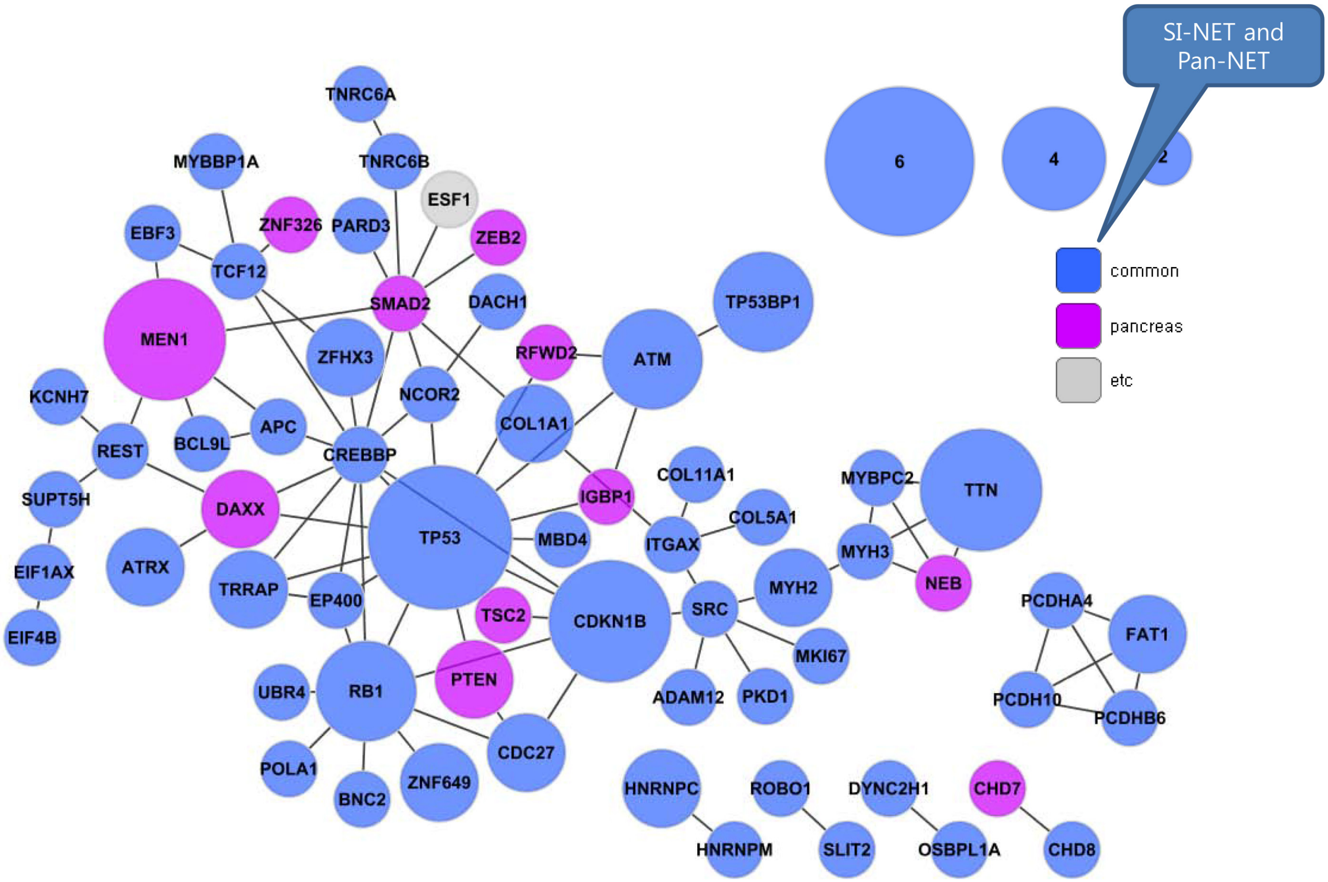

Supplementary Figure S4: Interaction networks derived from the mutated gene set. Each color was assigned by tumor-type occurrence.

Supplementary Table S1: Independent pathologic review

Case	Biopsy site	Architecture	Grade of tumor	Chromogranin	Synaptophysin	CD56	Others (+)
10–24099	Pancreas		3	NA	NA	NA	NA
09–40825	Colon	trabecular	2	NA	NA	NA	
09–29052	Liver		2	faint	(+)	(+)	CK-19 (+)
11–5361	Liver		3	(−)	(+)	(−)	
11–9317	Liver		3	(+)	(+)	(−)	
11–34268	Liver	trabecular	2	NA	NA	(+)	CK-7 (+)
11—39762	Liver		1	(+)	(+)	(+)	CK-19 (+)
11–43684	Pancreas		1	(+)	(+)	NA	
10–3204	Small intestine	trabecular	1	(+)	(+)		S100 (-)
10–29639	Colon	acinar	1	NA	NA	NA	
11–1661	Small intestine		3	(−)	(+)	(+)	CK-7 (−)<br>CK-20 (−)
11–4796	Stomach		1	(−)	(+)	NA	
11–16555	Pancreas	acinar	2	(+)	NA	NA	
